# Application of chromosomal microarray analysis in products of miscarriage

**DOI:** 10.1186/s13039-018-0396-y

**Published:** 2018-08-17

**Authors:** Xiangyu Zhu, Jie Li, Yujie Zhu, Wanjun Wang, Xing Wu, Ying Yang, Leilei Gu, Yuanyuan Gu, Yali Hu

**Affiliations:** 1 Department of Obstetrics and Gynecology, Nanjing University Medical School Affiliated Nanjing Drum Tower Hospital, Nanjing, 210008 Jiangsu China; 2 Department of Obstetrics and Gynecology, Nanjing Drum Tower Hospital, the Affiliated Hospital of Nanjing University Medical School, Nanjing, 210008 Jiangsu China

**Keywords:** Chromosomal microarray analysis, Miscarriage, Spontaneous abortion, Microarray, Genetic testing

## Abstract

**Background:**

Chromosomal abnormality is one of the major cause of spontaneous abortion. Most available guidelines suggest genetic testing after three miscarriages, which has been proved to be difficult to adhere to and somewhat of low cost-effectiveness. As chromosomal microarray analysis has been recommended to be applied on miscarriage products, we managed a retrospective study on our experience investigate the potential impact of this technique on previous guidelines and our present management on miscarried couples and products.

**Results:**

Of the 405 products of conceptions, the overall detection rate of pathogenetic results was 55.3% (224/405), including 7.1% (16/224) copy number changes which could be missed by conventional karyotyping analysis. Of the 222 natural conception samples, abnormal genetic results were found in 126 cases (56.8%). The detection rate in the assistant reproductive treatment group was 53.6% (98/183). No significant difference was found between these two groups (*p* = 0.645, *OR* = 1.110 with 95% CI: 0.713–1.726). The detection rate was 53.2% (75/141) in 141 product-of-conceptions (POCs) of mothers with adverse pregnancy histories. Of the 264 POC samples of mothers without abnormal pregnancy histories, 56.4% (149/264) were genetically abnormal. The detection rate and maternal age between these two groups were all compatible.

**Conclusions:**

Chromosomal microarray testing should be referred to couples at their first miscarriage regardless of the way how they get pregnant.

## Background

Pregnancy loss at any gestational age could be a catastrophic incident for the woman and her family. Seeking the potential cause is an effective way to estimate the recurrent risk and to take precautions to avoid another pregnancy loss. Though not fully deciphered, a good bunch of etiologies have been established to be related to pregnancy loss, among which, chromosomal abnormalities or genetic imbalances of the embryos or fetus have long been recognized as one of the major cause of miscarriage (< 20 weeks’ gestation). However, established in cytogenetic ages, most available practical guidelines mainly suggest genetic testing on a third miscarriage [2, 11, 23]. Even the most recent guidelines did not change the opinion [26].

With traditional cytogenetic techniques, culture failure due to microbial contamination and tissue inactivation was reported as high as 21–25% when applied to the product-of-conception specimens [28]. Maternal cell contamination and selected cell line proliferation might distort the real condition of fetal karyotype [20]. Resolution and potentially subjective judgment of the chromosomal alternation is another issue of this technique. All these drawbacks might be the handicap of the application and be recommended to a “recurrent” miscarriage.

However, due to the anxiety of the patients suffering from the miscarriage and other reasons, some investigations revealed that adhesion to guidelines was not ideal for both patients and professionals [12, 29, 30]. On the other hand, psychological stress and depression caused by miscarriage might be otherwise a high risk factor of miscarriage [17, 19, 31]. Thus we are considering if there’s something we could be done to improve the situation.

Thanks to the dramatic improvement in genetic testing techniques and reduced cost, we are now able to detect submicroscopic imbalances on a whole genome scale at a much lower price. Chromosomal microarray analysis (CMA) is one of the most prestigious technique. As a DNA-based technique, CMA is free form the drawbacks of traditional cytogenetic testing. What’s more, as CMA could reveal copy number changes over 400 kb (depending on different platforms that applied) in the scale of whole genome simultaneously, findings of submicroscopic copy number variations (CNVs) were reported as another advantage over traditional techniques. Levy B [16] found 1.6% pathogenic CNVs in their cohort. Other researchers found an additional 5.7–13% pathogenic CNVs in karyotypingly normal product-of-conception (POC) specimens [10, 22, 33]. As a result, CMA was generally accepted as a powerful technique to detect genetic imbalances in POC samples.

In this study, by reviewing our microarray data, we are going to confirm the effectiveness of CMA on POC samples, and further investigate the potential impact of this technique on previous guidelines and our present management on miscarried couples and products.

## Results

### Subject features and general findings

Of the 405 products of conceptions, gestational ages ranged from 5 weeks to 19 weeks and 6 days. Three hundred and sixty-seven (367/405,90.6%) POC samples were before 12 weeks and 6 days, or early miscarriage. Thirty-eight (38/405, 9.4%) POC samples were obtained from demised fetus between 13 gestational weeks and 19 gestational weeks and 6 days, also called late miscarriage. Maternal ages of these products-of-conception samples ranged from 20 to 46 (31.33 ± 4.74) years old.

The overall detection rate of pathogenetic findings was 55.3% (224/405). Maternal ages of the genetically abnormal POC samples were significantly older than the normal group (*p* < 0.001, see Table 1). Single aneuploidies were the most common abnormality, accounting for 77.2% (173/224) of all the abnormalities, among which, the most affected chromosomal aneuploidies were trisomy-16, trisomy-22 and monosomy X, accounting for 14.7% (33/224, including two cases of trisomy-16 with microscopic CNVs), 12.9% (29/224) and 11.6% (26/224) respectively (Table 2). CNV was the fourth most common abnormal finding, accounting for 7.1% (16/224) of the total abnormality (Table 2). Notably, CNV mentioned in this paper is not necessarily submicroscopic. Partial trisomy or monosomy that might be difficult to figure out under microscope was also defined as CNV (Table 4).

**Table 1:** Maternal age in different groups

	IVF	NC	Without APHs	With APHs	Normal	Abnormal
Maternal age	32.30 ± 4.68	30.51 ± 4.47	31.56 ± 4.93	30.91 ± 4.40	30.42 ± 4.30	32.00 ± 4.98
*p*-value	< 0.001		0.19		< 0.001	

*IVF* in vitro fertilization, *NC* natural conception, *APHs* adverse pregnancy histories

**Table 2:** Detailed spectrum of genomic imbalances in products of conception

Genomic imbalances			ART	NC	Without APHs	With APHs		13–19 + 6GWs	<13GWs	Total
						A	B			
Single aneuploidy	Autosomal trisomy	2	2	3	5	0	0	0	5	5
		3	0	2	2	0	0	1	1	2
		4	1	3	1	1	2	0	4	4
		5	1	0	1	0	0	0	1	1
		6	0	1	0	1	0	0	1	1
		7	3	3	5	1	0	0	6	6
		8	2	3	4	1	0	0	5	5
		9	1	1	2	0	0	0	2	2
		10	1	1	2	0	0	0	2	2
		11	1	1	1	0	1	0	2	2
		12	1	1	1	1	0	0	2	2
		13	1	9	3	5	2	1	9	10
		14	0	1	0	1	0	0	1	1
		15	6	5	6	3	2	0	11	11
		16^a^	15	18	19	4	10^a^	1	32	33
		18	3	4	4	1	2	3	4	7
		20	3	4	5	1	1	0	7	7
		21	6	6	11	1	0	2	10	12
		22	13	16	23	3	3	1	28	29
	Other aneuploidy	monosomy 21	3	1	4	0	0	0	4	4
		monosomy X	13	13	17	3	6	5	21	26
		XXY	0	1	0	0	1	0	1	1
CNV		CNV	7	9	11	2	3	1	15	16
Multiple aneuploidy		Double trisomy	7	3	7	2	1	0	10	10
		Trisomy 22 with monosomy X	2	0	2	0	0	0	2	2
Triploidy	triploidy	triploidy	3	10	9	3	1	3	10	13
	Hyper-triplody	Triploidy with tetrasomy X	0	1	1	0	0	0	1	1
		Triploidy with tetrasomy 16	0	1	1	0	0	0	1	1
		Triploidy with tetrasomy 8 and 14	0	1	0	0	1	0	1	1
	Hypo-triplody	Triploidy with disomy13	0	2	0	1	1	0	2	2
UPD		UPD	0	2	0	2	0	0	2	2
Mosaicism		Mosaic trisomy 6	1	0	1	0	0	0	1	1
		Trisomy 16 with mosaic trisomy 13	1	0	1	0	0	0	1	1
		Mosaic trisomy 18	1	0	0	1	0	0	1	1
Total abnormal			98	126	149	38	37	18	206	224

A: subgroup A, POC samples of women who experienced more than two previous pregnancies with adverse outcome; B: subgroup B, POC samples of women who experienced one adverse pregnancy history

*ART* assisted reproductive treatment, *NC* natural conception, *APHx* adverse pregnancy history, *GW* gestational week, *CNV* copy number variance, *UPD* uniparental disomy

^a^ two cases were trisomy-16 with CNVs

### Genetic findings in subgroups

According to the way how the women got pregnant at “this” time, we divided the samples into two groups: products of natural conception (NC) and products of assisted reproductive technique (ART). Of the 222 NC samples, abnormal genetic results were found in 126 cases (56.8%), the mean maternal age in this group was 30.51 ± 4.47 years old. The detection rate in the ART group was 53.6% (98/183) (Table 3). Mean maternal age of the ART group was 32.30 ± 4.68, significantly older than the NC group mother. When adjusted by the maternal age, the detection rate showed no significant difference between these two groups (*p* = 0.645, *OR* = 1.110 with 95% CI: 0.713–1.726). Among the ART group, 17 cases were products of intracytoplasmic sperm injection (ICSI) and 166 cases were products of in vitro fertilization (IVF). The detection rates were comparative between ICSI and IVF group (10/17 Vs 88/68, *p* > 0.5).

**Table 3:** Genetic abnormal findings in different groups

	ART	NC	Without APHx	≥3 APHx	With 2 APHx	<13GWs	13–19 + 6GWs
Abnormal	98	126	149	38	37	206	18
Normal	85	96	115	35	31	161	20
*p*-value (95%CI)	0.578 (0.604–1.326)		0.294			0.301 (0.728–2.776)	

According to pregnancy histories, we categorized the samples into two groups, with adverse pregnancy history (APHs) and without APHs. According to times of APHs, we further divided this group into two groups. One group included the POC samples of women who experienced more than two previous pregnancies with adverse outcome (indicated as subgroup A), the other included POC samples of women with only one adverse pregnancy (indicated as subgroup B). As a result, 141 cases were POCs of mothers with APHs. The detection rate was 53.2% (75/141) in this whole group, with 52.1% (38/73) and 54.4% (37/68) detection rate for subgroup A and B respectively (Table 3). Of the 264 POC samples of mothers without APHs, 56.4% (149/264) were genetically abnormal. The detection rate and maternal age between these two groups were all compatible (Table 1 and Table 2). No difference exists between either two groups (Table 3).

Two hundred six abnormal results were found in miscarriages before 12 weeks and 6 days, accounting for 56.1% cases (206/367) of this early pregnancy loss group. 47.3% (18/38) POC samples of 13 to 19 weeks and 6 days of gestational age were abnormal. Pathogenic CNVs were found in 4.1% (15/367) early miscarriages and 2.6% (1/38) in late miscarriages (Table 2). None of these detection rates showed significant differences between comparable groups.

## Discussion

### Additional yields support the use of CMA for POC samples

On chromosomal level, CMA results are highly accordant to cytogenetic results, and with a higher report rate [16]. What’s more, an additional 5.7–13% pathogenic CNVs in karyotypingly normal POC specimens [10, 22, 33]. Based on a more than 2000 cohort study, Dr. Levy and his colleagues provided a level III evidence to support the use of CMA for the cytogenetic evaluation of miscarriage specimens [16]. According to our study, we detected 5 cases with CNVs (1.2%) that less than 10 Mb (Table 4: case 12–16). This detection rate of submicroscopic CNVs is compatible to previous reports [10, 22, 33].

**Table 4:** CNVs found in pregnancy loss

No.	MA	GW	ART	APHx	CMA result	Size (Mb)
1	43	6	Y	N	arr [hg19] 8p23.3p11.1 (158,048-43,824,035) × 1, 8p11.1q24.3 (43,837,098-146,295,771) × 3,	43.7, 102.5
2	29	7	N	N	arr [hg19] 7p22.3p11.2 (162,702-57,780,598) × 3, 17p13.3p11.2 (525–21,518,996) × 1	57.6, 20.99
3	36	6	Y	2	arr [hg19] 8p23.3p12 (158,048-30,234,334) × 1, 8q22.1q24.3 (97,466,303-146,295,771) ×2–3	30, 48.8
4	28	8	N	3	arr [hg19] 19q11q13.43 (28,271,417-58,956,816) ×3	30.7
5	27	11	Y	N	arr [hg19] 8p23.3p12 (158,048-29,402,007) × 1, 8p12 (30,393,410-34,277,594) × 3	30, 3.88
6	27	8	Y	N	arr [hg19] 18p11.21q11.2 (12,520,909-19,043,748) × 3, 18q21.2q23 (52,653,009-78,013,728) × 1, 19q13.41q13.43 (54,872,973-58,956,816) × 3	6.5, 25.4, 4.1
7	33	6	Y	N	arr [hg19] 15q25.3q26.3 (87,014,450-102,429,040) × 1	25.4
8	22	7	N	N	arr [hg19] 11q22.1q24.2 (102,024,970-127,356,904) × 3, 11q24.3q25 (128,670,114-134,937,416) × 1	25.33, 6.267
9	33	7	N	3	arr [hg19] 1p36.33p36.11 (849,466-24,454,688) × 3, 7q36.2q36.3 (153,468,186-159,119,707) × 1	23.6, 5.65
10	29	7	Y	2	arr [hg19] 10q21.2q22.2 (61,408,306-76,588,258) × 1	15.18
11	25	5	N	N	arr [hg19] 18p11.32p11.21 (136,227-15,099,116) × 1	14.96
12	30	6	N	N	arr [hg19] 1p36.33p36.22 (849,466-10,391,536) × 1	9.5
13	30	11	N	N	arr [hg19] 10q22.3q23.2 (81,630,468-88,785,190) × 3	7.16
14	21	10	N	2	arr [hg19] 22q11.21 (18,648,855-21,800,471) × 1	3.1
15	25	12	Y	N	arr [hg19] 22q11.21 (18,916,842-21,163,516) × 3	3.1
16	29	9	Y	N	arr [hg19] 22q11.22q11.23 (22,331,458-23,652,518) × 3	1.32

*MA* maternal age, *GW* gestation week, *ART* assisted reproductive technique, *APHx* adverse pregnancy history, *Y* yes, *N* no

Moreover, CMA may be more sensitive and accurate to detect imbalanced chromosomal rearrangement. POC, as a sort of proband, could be used as a perfect clue to uncover the potential existence of a balanced translocation in either of the parent. According to our CMA results, we deduced that 5 miscarriages may be consequences of recombination of balanced translocations or inversions in either of the parents (Table 4: case 2, 3, 5, 6 and 9). Two cases might be caused by complex chromosome rearrangement (Table 4: case 1 and 8). Other 4 miscarriages might be due to de novo mutation or mosaic conditions of their parents (Table 4: case 4, 7, 10 and 11). As these 11 cases might not be effectively recognized under microscope for the concealed nature of these chromosomal change, we grouped them as CNVs together with the previous 5 truly CNV cases to highlight the potential advantages of CMA.

In our present data, 2 UPD cases were revealed. The ability to detect uniparental disomy (UPD) with single nucleotide polymorphism (SNP) markers is another advantage of the microarray method we have used. As risk of rare imprinting disorders raises after ART [27], microarray could be applied as a useful tool to make further research.

### Pregnancy loss and adverse pregnancy history

Many researchers suggested that a history of trisomy conception was a risk factor of future pregnancies being trisomic, especially for women younger than 35 years old [1, 5, 9, 32]. A higher risk of nondisjunction was thought to be existed in these women [32]. However, according to our study, no difference of genetic abnormal rate was found between either two groups of patients without APHs, one adverse pregnancy and more than one adverse pregnancy.

Miscarriage is a multifactorial situation. In a large cohort study, Bhattacharya suggested that the risk of a further miscarriage increased in women who had one and two miscarriages regardless of the etiology [4]. Researchers have also found that women with recurrent pregnancy loss (RPL) suffered from more psychological stress and depression [7, 15, 19], which could trigger a negative feedback [8] threatening subsequent conception [17, 19, 31].

Bernardi and her colleagues’ research [3] revealed that selective RPL evaluation based upon chromosome testing was a cost-saving strategy when compared to universal RPL evaluation. As a further supportive evidence of this opinion, our CMA result have found 2 cases (Table 3: case 4 and 5) of genetic imbalance which was quite possibly be the consequence of recombination of a balanced translocation existed in either parent. The detection of this translocation could be finely defined as the etiology of potential recurrent miscarriage [24, 25].

Considering the benefits of CMA, the impact of spontaneous miscarriage on subsequent pregnancy and the potential effect-economic benefits, we suggest that CMA test on POC-sample should be recommended to couples who suffered from their first miscarriage.

### Assisted reproductive technique versus natural conception

ART is a major therapy method in many infertile couples. Only a few studies with limited sample volume were concerned on the risk of chromosomal abnormalities after ART treatment. And to some pitfalls, the results were still controversial. Campos-Galindo et al. [6] tested 189 samples from ART or NC pregnancies with KaryoLite™ BoBs™, and they observed a considerably higher rate of aneuploidy in the ART group using the patient’s own oocytes. However, the maternal age of this group was significantly higher. Some detailed studies found no increased risk of chromosomal abnormalities due to ART [14, 18]. A recent meta-analysis had come to the same conclusion that no statistical difference was existed in risk of chromosomally abnormal miscarriage between ART and NC groups [21]. Our result as well indicated no increased burden of genetic abnormalities occurred after ART (data were adjusted by maternal age). These studies might relieve us a bit of the worries about ART. However, as limited by the sample size, we didn’t further divide them according to specific technique of ART they applied. More samples are need to make a conclusive result.

## Conclusion

According to our experience and of previous researches, CMA presented as reliable and comprehensive technique to detect genetic imbalances in POC samples and stillbirths. As no significant difference was found in ART group versus NC group and pregnancy with APHs versus without APHs, genetic testing should be reoffered to couples at their first miscarriage, and couples undergo ART could be a little relieved of the additional risk of chromosomal abnormalities.

## Methods

### Materials

From January 2014 to November 2017, reportable results were obtained from 405 POCs, including villi (380 cases) and tissues of fetus while available (25 cases). All the samples were collected routinely when parents wanted to seek a genetic etiology of the miscarriages. Consent forms were signed by couples before the tests. Parental peripheral blood specimens were obtained together with the products of conceptions. History of previous pregnancy (s) and the way how they got conceived (natural conception or by assisted reproductive techniques) at this time were recorded. Adverse pregnancy history (APHs) was defined when either condition existed as below: spontaneous miscarriage or stillbirth; termination of pregnancy because of fetal malformation; infantile death because of severe structural abnormalities.

### Methods

All the villi were carefully separated by needles under the anatomical microscope. Any macroscopic blood was washed away from tissues by saline solution. By using four highly polymorphic short tandem repeats, D2S1338, D7S820, D13S317 and D21S11 for specific, maternal cell contamination was ruled out for all the 405 samples.

Chromosomal microarray analysis was performed using the Affymetrix CytoScan platform, which contains both SNP markers and copy markers. All genomic DNA samples were digested, amplified, fragmented, labeled and hybridized to CytoScan 750 K chips according to the manufacturer’s protocol. Raw data was analyzed by ChAS 3.1 software (Affymetrix, USA). Interpretation of the CNVs defined according to the ACMG guidelines [13]. CNVs of unknown significance were further tested on the parents’ DNAs by quantitative fluorescent PCR. If it was constitutive in phenotypically normal parent, the CNV of unknown significance was never thought to be causative and then classified as “normal” in this study.

### Statistical analyses

Statistical analysis was performed using IBM SPSS Statistics, version 21. T-test, regression analysis and Chi-square test were applied in necessary situations. *p* < 0.05 was considered statistically significant.

## Abbreviations

APHs: Adverse pregnancy history
ART: Assisted reproductive technique
CMA: Chromosomal microarray analysis
CNV: Copy number variations
GW: Gestational age
ICSI: Intracytoplasmic sperm injection
IVF: In vitro fertilization
MA: Maternal age
NC: Natural conception
POC: Product-of-conceptions
RPL: Recurrent pregnancy loss
SNP: Single nucleotide polymorphism
UPD: Uniparental disomy
